# The impact of resource competition on neurite outgrowth

**DOI:** 10.1186/1471-2202-12-S1-P353

**Published:** 2011-07-18

**Authors:** Johannes JJ Hjorth, Jurjen Broeke, Huib Mansvelder, Jaap van Pelt, Arjen van Ooyen

**Affiliations:** 1 Integrative Neurophysiology, Center for Neurogenomics and Cognitive Research (CNCR), VU University Amsterdam, De Boelelaan 1085, 1081 HV Amsterdam, The Netherlands; 2 Functional Genomics, Center for Neurogenomics and Cognitive Research (CNCR), VU University Amsterdam, De Boelelaan 1085, 1081 HV Amsterdam, The Netherlands

## 

Axonal and dendritic growth is a highly dynamic process, where neurites elongate, bifurcate and retract. Local calcium influx [1] or local depolarisation [2] of a neurite can cause it to grow faster. The rapid growth of one branch is often accompanied by a retraction of other nearby branches [3]. This process is important for correct wiring of neuronal circuits [4]. In this study we investigate resource competition as a potential mechanism to explain the interactions between neurite branches. We have created a full compartmental model developed in python [5] using numpy [6] building on a previous modelling study [7] which suggested that the interplay between neurites might be explained by competition for resources produced in the soma; however, this has not been rigorously investigated. Neurons are stabilised by a cytoskeleton of microtubules [8]. Tubulin is produced in the soma from where it moves through diffusion and active transport to the growth cone at the tip of the neurite. At the growth cone the tubulin is polymerised, and becomes part of the microtubule cytoskeleton. In our model, neurite elongation is driven by concentration dependent tubulin polymerisation at the growth cone. We show that tubulin competition can account for the interplay between elongating and retracting neurites seen in [1,3]. We quantify the distance dependence of the interactions between growth cones. Furthermore we show that the model can correctly predict the growth of an experimentally recorded growth cone given the information about how the neighbouring neurites grow out. We plan to make additional experiments to test our predictions and investigate whether the estimated values for the decay, diffusion and active transport required for competitive effects in the model match those of tubulin.
